# SiGerinn – interprofessional patient safety training in perinatal care: Concept and formative evaluation

**DOI:** 10.3205/zma001749

**Published:** 2025-04-15

**Authors:** Katharina Averdunk, Judith Hammerschmidt, Angela Klein, Matthias Weigl

**Affiliations:** 1 University Hospital Bonn, Institute for Patient Safety (IfPS), Bonn, Germany; 2 University Hospital of Saarland, Department of Nursing Administration, Homburg, Germany; 3 University Hospital Bonn, Clinic for Gynaecology and Gynaecological Oncology, Gynaecological Psychosomatics, Bonn, Germany

**Keywords:** interprofessional education (IPE), patient safety, interprofessional collaboration, collaborative practice, perinatal care

## Abstract

**Objectives:**

Interprofessional education (IPE) seeks to promote interprofessional competencies among care providers, to help improve quality of care and patient safety. Interprofessional collaboration (IPC) and teamwork are particularly important in perinatal care. Therefore, we introduced *SiGerinn (Safety and interprofessionality in perinatal care – Together and from the beginning)*, an interprofessional patient safety training for students in perinatal care. Here, we aim to present its concept and formative evaluation results.

**Methods:**

The training programme designed for midwifery, nursing and medical students, focuses on IPC core competencies and safety communication techniques pertaining to perinatal care. It consists of two one-hour training sessions implemented in the setting of an inter-professional training ward in the local postpartum care unit of an academic university hospital. Additionally, skilled practice trainers supervised practical application of learning contents in the ward. Formative evaluation involved semi-structured interviews with practice trainers, focusing on relevance and feasibility.

**Results:**

Practice trainers reported overall acceptance of the training content. High individual motivation was identified as a key success factor for implementing IPE and IPC. Leaders’ support and structural feasibility were also identified as critical factors. The remaining challenges for future training adaptations need to be considered for sustainable transfer to safe perinatal care practice.

**Conclusion:**

Our formative evaluation and preliminary experiences of *SiGerinn* revealed several critical success factors and barriers for IPE and IPC in perinatal care. We identified critical challenges and opportunities for future project refinement, serving as a blueprint for similar initiatives in perinatal care and beyond.

## Zusammenfassung

**Zielsetzung:**

Interprofessionelle Lehre (IPL) fördert interprofessionelle Kompetenz von Gesundheitsfachpersonen und damit die Versorgungsqualität und Patientensicherheit. Effektive interprofessionelle Zusammenarbeit (IPZ) und Teamarbeit sind insbesondere in der geburtshilflichen Versorgung von großer Bedeutung. Das Projekt *SiGerinn* (Sicherheit und Interprofessionalität in der Geburtshilfe – Gemeinsam und von Beginn an) beinhaltet ein interprofessionelles Training zum Thema Patientensicherheit in der geburtshilflichen Versorgung. Dieser Projektbericht präsentiert das Konzept und Ergebnisse der formativen Evaluation.

**Methodik:**

Der Fokus des Patientensicherheitstrainings für Studierende der Hebammenwissenschaft und Medizin sowie Auszubildende der Gesundheits- und Krankenpflege ist die Vermittlung von Kernkompetenzen der IPZ und Kommunikationstechniken im Kontext Geburtshilfe. Das Training umfasst zwei einstündige Trainingseinheiten, durchgeführt in Zusammenarbeit mit einer interprofessionellen Ausbildungsstation auf der Wöchnerinnenstation eines Universitätsklinikums. Qualifizierte Praxisbegleitende unterstützen die praktische Anwendung der Lerninhalte. Die formative Evaluation mit dem Fokus auf Relevanz und Umsetzbarkeit erfolgte auf der Basis von teilstrukturierten Interviews mit den Praxisbegleitenden.

**Ergebnisse:**

Die Praxisbegleitenden berichteten über eine allgemeine Akzeptanz der Trainingsinhalte. Eine hohe individuelle Motivation wurde als entscheidender Erfolgsfaktor für die Umsetzung von IPL und IPZ identifiziert. Die Unterstützung durch Leitungspersonen und die strukturelle Umsetzbarkeit wurden ebenfalls als kritische Faktoren hervorgehoben. Anpassungen des Trainingskonzepts müssen strukturelle Herausforderungen berücksichtigen, um die nachhaltige Implementierung in eine sichere Versorgungspraxis in der Geburtshilfe zu gewährleisten.

**Schlussfolgerung:**

Die formative Evaluation und die bisherigen Erfahrungen im Projekt *SiGerinn* haben entscheidende Erfolgsfaktoren und Barrieren für IPL und IPZ in der geburtshilflichen Versorgung aufgezeigt. Es konnten wesentliche Herausforderungen und Möglichkeiten für Anpassungen des Projekts identifiziert werden, die als Anhaltspunkte für ähnliche Initiativen in- und außerhalb der Geburtshilfe dienen können.

## 1. Introduction

Collaborative practice is defined as *“[..] when multiple health workers from different professional backgrounds work together with patients, families, carers and communitie”* [1], promoting high-quality care and patient safety [1], [2], [3]. Perinatal care embodies particularly critical demands for collaborative practice. First, shared responsibilities between midwives and obstetricians and differences in professional cultures might hinder collaboration [4], [5], [6], [7]. Second, parturient women differ from other patient groups owing to the unique nature of childbirth, combining physical and emotional aspects, with the fear of rare but potentially severe complications [4], [8]. Effective interprofessional collaboration (IPC) is crucial to prevent fetal and maternal harm, both physical and psychological [4], [8]. National and international health policies also emphasise the importance of collaboration in perinatal care [9], [10], [11].

Interprofessional education (IPE) is learning *about, from* and *with* other professional groups [1]. IPE aims to promote effective IPC in order to improve care outcomes [1], [3], [12]. However, IPE is not yet a regular part of health profession curricula [1], [13], [14], mainly due to structural barriers such as conflicting schedules [13], [15], [16]. In perinatal care, attending different, just occasionally overlapping training programs might result in varying perspectives on pregnancy and childbirth among the different professional groups [4], [7], [15]. This structural gap in education might aggravate potential barriers to interprofessional team performance [1], [6], [14].

Existing projects on IPC predominantly focus on rather technical competencies such as emergency skills [15], [17], [18]. This does not fully capture the high relevance of communication and teamwork challenges in everyday (perinatal) care [16]. Although there is a positive trend [8], [19], [20], only a few IPE approaches explicitly target safety-relevant topics in IPC. A recent study in Germany reported positive effects of communication and team training on patient safety in perinatal care [8].

We conceived our project called *SiGerinn* (German: Sicherheit und Interprofessionalität in der Geburtshilfe – Gemeinsam und von Beginn an; *Safety and interprofessionality in perinatal care – together and from the beginning*) and aimed to develop, implement and evaluate an inter-professional patient safety training for students from different professions in perinatal care. The training focuses on IPC and communication among team members and with patients. To the best of our knowledge, this is the first report on an IPE program to promote patient safety and IPC for students in perinatal care in Germany.

This report aims to present our concept, IPE content, format and first implementation experiences to the community. Drawing upon formative evaluation results, we sought to describe the overall acceptance, feasibility and challenges to the sustainability of our IPE training project. 

## 2. Project description

### Concept development: Learning objectives, format and implementation

The *SiGerinn* project was collaboratively developed by an interprofessional team of individuals involved in patient safety, nursing and obstetric care. Throughout the development process and project implementation, psychologists, physicians, nurses and midwives were actively involved to ensure a comprehensive approach. The target learner groups were medical, midwifery and nursing students undergoing their practical studies in postpartum care. Medical students were in their final, practical year of a six-year curriculum, while midwifery and nursing students were required to have completed at least three out of eight (midwifery) or six (nursing) study semesters.

**Figure 1:**
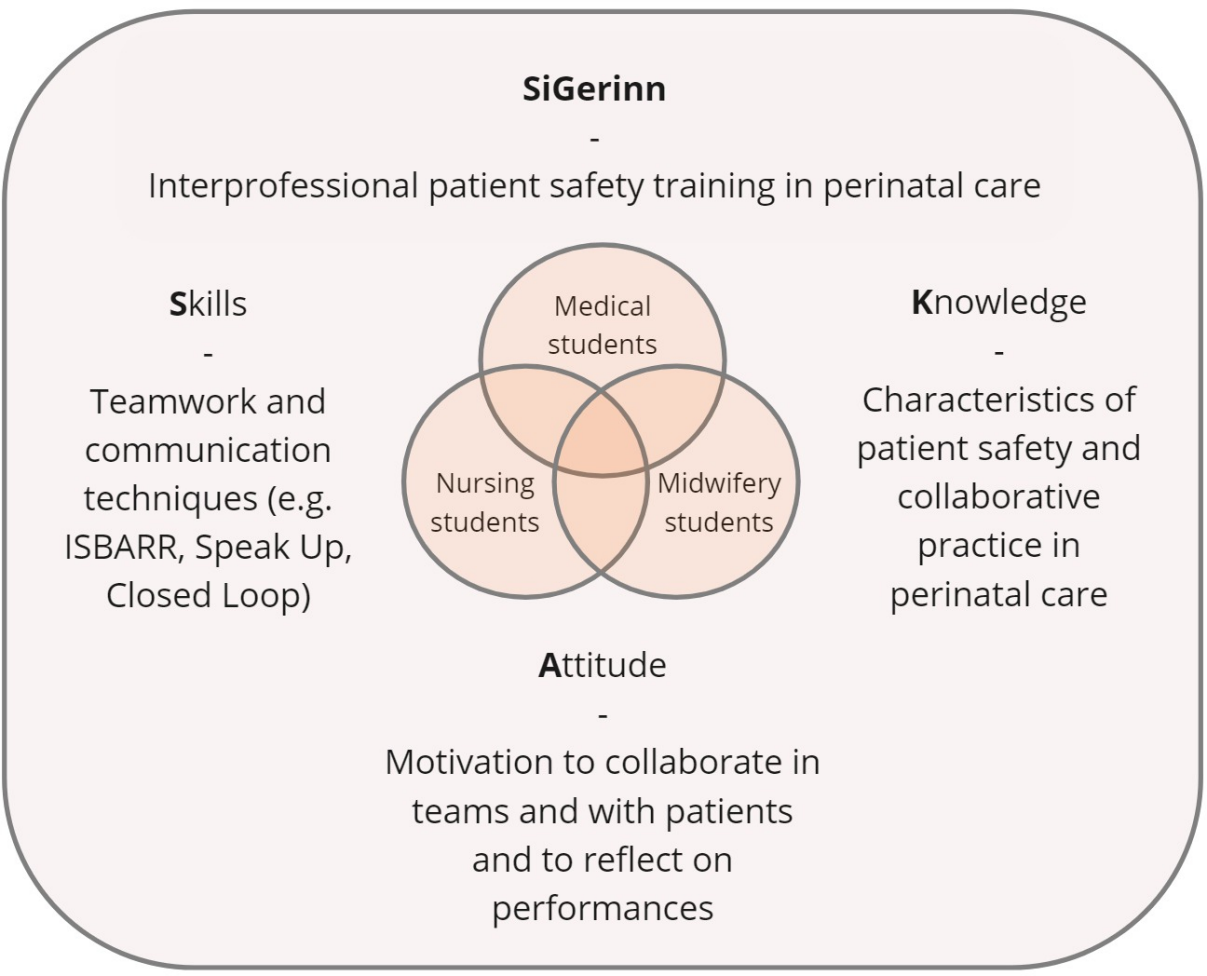
SiGerinn – concept and key contents

 For the essential competencies for training, we referred to the Canadian *National Interprofessional Competency Framework* [3], supplemented by the *Core Competencies for Interprofessional Collaboration* [21]. The suitability and applicability of these competencies to perinatal care were carefully considered using the *Conceptual Model of Midwife-Physician-Collaboration* [6] as well as findings from Avery et al. [15] and Cornthwaite et al. [4]. The competencies for training in the *SiGerinn* project were role clarity in perinatal care, teamwork and communication skills, interprofessional conflict resolution and patient-centredness. The respective learning objectives were defined based on Baker’s KSA model which classifies teamwork mechanisms into knowledge, skills and attitude [22]. Teamwork and communication tools were based on the TeamSTEPPS approach [23]. Figure 1 (Fig. 1) shows a schematic illustration of the concept and core content of *SiGerinn*. 

The training consisted of two one-hour training sessions conducted in the ward and a third reflection session which included feedback and joint discussion on transferring theoretical learnings to everyday work. To promote interprofessional learning, we aimed to include one student from each profession (medicine, midwifery and nursing) in each training cycle. To facilitate future practical application, participants received “pocket cards” (pocket-sized laminated cards) for daily use, showing the acquired communication techniques. Table 1 (Tab. 1) provides details on training procedures. 

For the inaugural implementation, we collaborated closely with the team of the “interprofessional training ward” at the local postpartum care unit (German: Wöchnerinnen – Interprofessionelle Ausbildungsstation, W-IPSTA), the first of its kind in a perinatal care setting in Germany [24]. *SiGerinn* was an independent, complementary part of the theoretical training program for W-IPSTA participants, focusing explicitly on contents related to patient safety. The practical application in the ward was supervised by skilled practice trainers in W-IPSTA, including a gynaecologist, a midwife and a nurse. 

### Evaluation

#### Objective

The overall aim and procedure of the project evaluation were twofold: First, a formative evaluation addressed the relevance and feasibility of the training content, according to the in-charge practice trainers. Relevance included overall acceptance and added value, while feasibility comprised the applicability of the learning content and sustainability of the project. Evaluation results will be used to guide subsequent adaptations and refinements within the *SiGerinn* project, ensuring a cycle of continuous improvement and optimisation. Second, an ongoing pre-/post-evaluation among project participants, i.e. medical, midwifery and nursing students, will examine their competency development and perceptions of the relevance of IPE during studies (to be completed in December 2024). This report solely focuses on the formative evaluation (aim 1). Before starting, all evaluation steps were approved by our local ethics committee (#203/22). For formative evaluation, the following research question was formulated: What are the perceptions of practice trainers regarding the implementation of the *SiGerinn* project, specifically in terms of its relevance and feasibility for acquiring and applying interprofessional competencies among participating students?

#### Data collection

We designed semi-structured interviews with practice trainers who were actively involved in the project on-site. All participants provided informed consent prior to the interviews. The interview guide was developed to explore the practice trainer’s reflections on the project’s concept and implementation. 

#### Data analysis

Qualitative content analysis was performed to examine the interview data [25]. Interview statements were analysed via identification of deductive categories capturing the key objectives of interest for the project evaluation, i.e. relevance and feasibility.

## 3. Results

Our project *SiGerinn* was successfully implemented as part of the interprofessional training ward in the local postpartum care unit (W-IPSTA). As of February 2024, the collaborative programme of W-IPSTA and* SiGerinn* was provided six times, involving two medical students, five midwifery students and six nursing students. The on-site training sessions were conducted by a nurse or a midwife, both affiliated with the Institute for Patient Safety, University Hospital Bonn. 

The interview partners for formative evaluation were three practice trainers in W-IPSTA: a gynaecologist, a nurse and a midwife. To ensure anonymity within this group, this result section does not interlink with the interview fragments, neither with professions nor among each other. Quotes were translated from German to English by the authors. The statements were classified according to the categories relevance and feasibility. 

### Relevance

The formative evaluation showed overall acceptance and positive feedback on the *SiGerinn* training concept. The key aspects of the content that resonated with the participants were empowerment and engagement of patients, enhanced responsibility for patient safety and acquisition of standardised communication techniques such as patient handover according to ISBARR (*Introduction – Situation – Background – Assessment – Recommendation – Read back*) framework. The pocket cards for handout were perceived as particularly useful.

*Practice trainer (P): “I have always liked the trainings, because they were very concise. I thought that the provision of pocket cards as a background material was great”.*

*P: “There is already awareness of safety issues [among students] [...]. The training reinforces it again and again”.*

Moreover, interview partners mentioned that they might have already instinctively applied specific communication techniques, such as ISBARR and briefing or debriefing, in their daily practice before the training without being aware of the formal names or structures.

*P: “Some people knew it [the communication techniques] from practice […]. In retrospect, we were able to name what we had already been doing intuitively or based on experience and knowledge”.*

### Feasibility

Interviewees highlighted that organisational and individual factors impacted the applicability of the learning content. Students needed time and clear structures to embed acquired knowledge into care routines. 

*P: “Using a standardised method [for patient handover] can help internalise this scheme. Then it works”.*

However, interviewees identified conflicting schedules as a significant barrier to effective interprofessional learning among students from diverse professional groups. Moreover, the motivation of the individual students was highlighted as critical for the practical use of the acquired competencies. 

*P: “When we worked highly focused on the interprofessional training ward, we were able to use the methods. But, the motivation of participants is very important”.*

*P: “Being motivated to apply the methods is crucial”.*

Practice trainers emphasised high motivation among students to apply new knowledge and to work effectively in interprofessional teams. Awareness of their role, opportunities to introduce changes and courage to follow their concepts were additional facilitators.

Moreover, practice trainers reflected upon their own role and perceived it as an important facilitator for the practical application of learning content among students. 

*Interviewer: “Do you think students transfer the content into practice?”;*

*P: “When we suggest it”.*

*P: “When I only know a method but there is nobody to motivate, to help me apply it again and again, then I will probably stop [to use the methods]”.*

*P: “From my perspective, as a practice trainer, I see myself as a facilitator”.*

Finally, the practice trainers stated that the students are likely to transfer safety knowledge and behaviour into care routine depending on opportunities to practice. 

*P: “When I have learned and practiced a method […] and have experienced benefits of using it […], then it [the training] will have an effect”.*

*P: “My impression is, they think, they will continue [to apply the learning content]. They recognise the great time and opportunity they had [in this programme]. I always hope that they have the courage to continue. That it does not disappear”.*

The practice trainers believed that the sustainability of this educational approach and the application of the training contents in practice heavily relied on the individual motivation of students, teachers and clinical leaders and the leaders’ support. According to one practice trainer, sustainable implementation of interprofessional methods in daily care routines requires standardised and mandatory procedures. 

*P: “It is doable [to apply the content], but certainly depends on the leader”.*

*P: “It depends on how many people know it. If there was a rule to use it and control mechanisms, then it will be done”.*

*P: “It needs to be a team effort; that everyone knows and uses it”.*

Lastly, the interviewees emphasised the relevance of engaging advocates such as team leaders, trained team members and former project participants to foster general acceptance of the project and its content.

*P: “If this should sustain, it needs to be one person in the ward who supervises this way of learning; a person who multiplies it in the team. It is much better anyway if it is a team member”.*

## 4. Discussion

Effective IPC is crucial for ensuring patient safety in perinatal care [4], [6], [8]. To address this, healthcare organisations and educators are now focusing on providing interprofessional training from the early stages of professionals’ careers. This report aims to describe the IPE project *SiGerinn*, its concept and the findings of its formative evaluation. The formative evaluation was based on practice trainers’ perspectives on the project’s implementation with a focus on the relevance and feasibility of the training content. The insights gained can help foster further improvement of IPE training and related projects – in and beyond perinatal care. 

To the best of our knowledge, this is the first report on an IPE intervention on patient safety for medical, midwifery and nursing students in perinatal care in Germany. This contribution is significant given the high prevalence of deficits in IPC as well as the devastating impact of communication failures on patient harm, both within and outside of perinatal care [2], [4], [7], [8], [11], [26], [27], [28]. Our IPE training approach addresses the pressing need for effective interprofessional communication and collaboration in high-stake safety settings. By aligning with educational measures in care environments that seek to promote IPC, our approach has the potential to improve patient outcomes and safety in various healthcare settings. 

Similar to related projects (e.g. [18], [20], [26], [29]), our formative evaluation results underscore the relevance and overall acceptance of IPE in IPC techniques, applied to a perinatal care setting. The insights gained and reported experiences can be used as a starting point for future IPE attempts in perinatal care. Moreover, our results corroborate high levels of motivation as a key success factor for IPE and IPC [19], [20], [29]. Furthermore, they point towards high motivation among students to work effectively in interprofessional teams. Regarding the current deficits in IPC and IPE, a pertinent question is how this initial motivation can be augmented to persist in future care practice. In this respect, our results emphasise the need to ensure the support of mentors and senior leadership in the clinic. This corroborates previous findings on key factors for successful IPE and IPC [4], [16]. 

Our current collaborative approach with the W-IPSTA team highlights the crucial role of practice trainers as facilitators in practical application and dissemination of learning content. Our findings emphasise their significance in ensuring a sustainable implementation of IPC. This aspect adds value to previously reported projects, as it not only evaluates the training contents but also examines its transferability of learning achievements to real-world practice. However, further research is required to validate this using exploratory studies designed to evaluate facilitators’ roles.

Finally, we confirmed previous findings that organisational constraints and conflicting structures among study programmes might impede the continuous implementation of IPE [13], [15], [16], also in perinatal care settings. This needs to be carefully considered for future adaptations within our project as well as in similar attempts.

### Limitations

Primary limitations include the narrow scope of the project and the limited sample size in our formative evaluation. Both these limitations may affect the generalisability of our results. 

As a pioneering initiative, our project *SiGerinn* involved a relatively small number of participants. Moreover, the duration of the training (two hours plus one reflection session) might be insufficient to drive sustained changes in practice. Hence, future initiatives should aim to increase the number of participating students, extend the training period and adopt a longitudinal training concept. Nevertheless, our close collaboration with the W-IPSTA program was a significant strength, as it enabled practice trainers to provide ongoing guidance and support, ensuring that learning extended beyond our training sessions. This helped facilitate a more effective transfer of knowledge into practice. 

Our formative evaluation involved critical insights from all three of the practice trainers. However, the small sample size may limit the ability to comprehensively capture the full range of perspectives needed to reflect on project implementation. Therefore, future evaluations of IPE projects should consider expanding the range of involved perspectives (e.g., including reports of involved students) and, ideally, set a longitudinal study design with follow-up assessments to evaluate long-term effects. 

Lastly, a significant challenge in evaluating IPE projects is the limited evidence base and inconsistency in outcome measures [28]. To address this, structural adjustments are necessary, such as embedding IPE in study curricula instead of small-scale projects [2], [13], [14], [20]. This alignment might enhance long-term benefits and bring about measurable improvements in patient safety. 

## 5. Conclusion

We introduced and successfully integrated interprofessional patient safety training into the “interprofessional training ward” in a local postpartum care unit. Our formative evaluation highlighted the importance of establishing training on IPC in perinatal care, identifying key success factors and barriers. We observed good acceptance of the project among the interviewed professionals and a high level of stakeholder motivation for IPC. Future attempts should address organisational support, particularly leaders’ support, and the feasibility and opportunities for a joint curriculum to maintain motivation among future health professionals to work effectively in interprofessional teams. Lastly, we suggest collaborating with practice facilitators in the context of IPE and IPC projects on patient safety.

## Authors’ ORCIDs

- Katharina Averdunk: [0000-0002-9383-0024]
- Judith Hammerschmidt: [0000-0003-4159-2121]
- Angela Klein: [0009-0005-0829-3770]
- Matthias Weigl: [0000-0003-2408-1725]

## Competing interests

The authors declare that they have no competing interests. 

## Figures and Tables

**Table 1:**
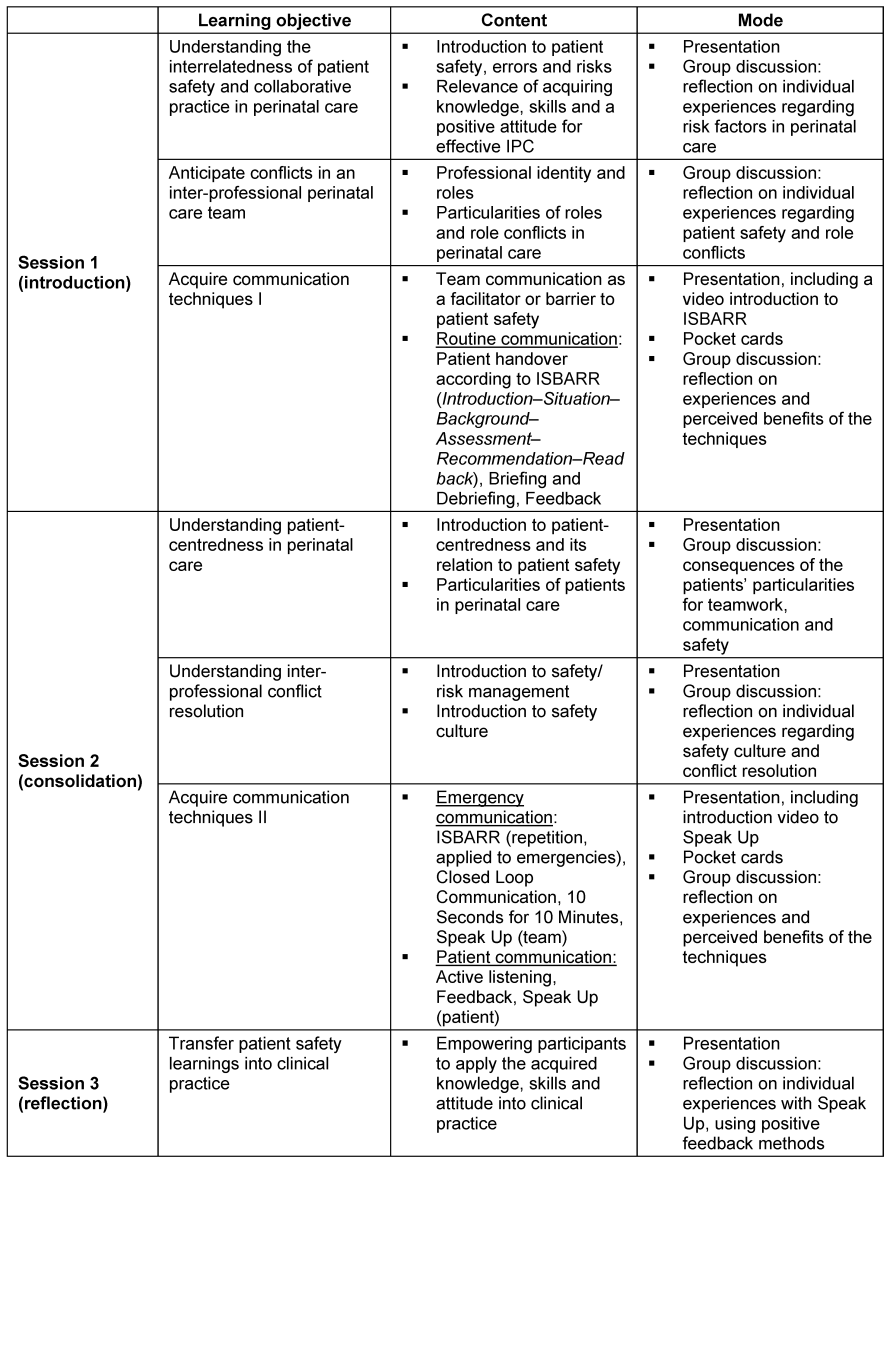
SiGerinn curriculum – content and implementation

## References

[1] Leonard M (2004). The human factor: the critical importance of effective teamwork and communication in providing safe care. Qual Saf Health Care.

[2] World Health Organization (2010). Health Professions Networks Nursing & Midwifery Human Resources for Health. Framework for Action on Interprofessional Education & Collaborative Practice.

[3] Canadian Interprofessional Health Collaborative (2010). CIHC-National-Interprofessional-Competency-Framework.

[4] Cornthwaite K, Edwards S, Siassakos D (2013). Reducing risk in maternity by optimising teamwork and leadership: an evidence-based approach to save mothers and babies. Best Pract Res Clin Obstet Gynaecol.

[5] Baecher-Lind L, Fleming AC, Bhargava R, Cox SM, Everett EN, Forstein DA, Madini Sims S, Morgan HK, Morosky CM, Royce CS, Sonn TX, Sutton JM, Graziano SC (2022). Enhancing interprofessional collaboration and interprofessional education in women's health. Med Educ Online.

[6] Smith DC (2015). Midwife-physician collaboration: a conceptual framework for interprofessional collaborative practice. J Midwifery Women Health.

[7] Schölmerich VLN, Posthumus AG, Ghorashi H, Waelput AJM, Groenewegen P, Danktaş S (2014). Improving interprofessional coordination in Dutch midwifery and obstetrics: a qualitative study. BMC Pregnancy Childbirth.

[8] Hüner B, Derksen C, Schmiedhofer M, Lippke S, Riedmüller S, Janni W, Reister F, Scholz C (2023). Reducing preventable adverse events in obstetrics by improving interprofessional communication skills - Results of an intervention study. BMC Pregnancy Childbirth.

[9] Bundesministerium für Gesundheit (2017). Nationales Gesundheitsziel. Gesundheit rund um die Geburt.

[10] World Health Organization (2021). Global Patient Safety Action Plan 2021-2030: Towards Eliminating Avoidable Harm in Health Care.

[11] Aktionsbündnis Patientensicherheit (2021). Thema zum Welttag der Patientensicherheit Unsicherheiten bei der Geburt, Geburtstraumata und schwere Komplikationen. Wenn man nicht sagen kann: „Sicher vom ersten Atemzug an“.

[12] Webster CS, Coomber T, Liu S, Allen K, Jowsey T (2024). Interprofessional Learning in Multidisciplinary Healthcare Teams Is Associated With Reduced Patient Mortality: A Quantitative Systematic Review and Meta-analysis. J Patient Saf.

[13] Walkenhorst U, Mahler C, Aistleithner R, Hahn EG, Kaap-Fröhlich S, Karstens S, Reiber K, Stock-Schröer B, Sottas B (2015). Position statement GMA Committee--"Interprofessional Education for the Health Care Professions". GMS Z Med Ausbild.

[14] Frenk J, Chen L, Bhutta ZA, Cohen J, Crisp N, Evans T, Fineberg H, Garcia P, Ke Y, Kelley P, Kistnasamy B, Meleis A, Naylor D, Pablos-Mendez A, Reddy S, Scrimshaw S, Sepulveda J, Serwadda D, Zurayk H (20104). Health professionals for a new century: transforming education to strengthen health systems in an interdependent world. Lancet.

[15] Avery MD, Jennings JC, Germano E, Andrighetti T, Autry AM, Dau KQ, Krause SA, Montgomery OC, Nicholson TB, Perry A, Rauk PN, Sankey HZ, Woodland MB (2020). Interprofessional Education Between Midwifery Students and Obstetrics and Gynecology Residents: An American College of Nurse-Midwives and American College of Obstetricians and Gynecologists Collaboration. J Midwifery Women Health.

[16] Handgraaf M, Wallin J, Groll C, Posenau A (2023). Identification of barriers and facilitators of successful interprofessional education (IPE) – a scoping umbrella review / Identifizierung der Einflussfaktoren für die interprofessionelle Ausbildung (IPE) – ein Umbrella Scoping Review. Int J Health Prof.

[17] Buljac-Samardzic M, Doekhie KD, van Wijngaarden JD (2020). Interventions to improve team effectiveness within health care: a systematic review of the past decade. Hum Resour Health.

[18] Agricola CJ, Juschka ML, Mohr S, Neumann FA, Zyriax BC (2023). Interprofessionelles Lernen von Studierenden der Hebammenwissenschaft und Humanmedizin: Das Lehr-Projekt „IPE-MidMed“.

[19] Howarth SD, Fielden SA, O'Hara JK (2022). How do we educate medical students interprofessionally about patient safety? A scoping review. J Interprof Care.

[20] Wipfler K, Hoffmann JE, Mitzkat A, Mahler C, Frankenhauser S (2019). Patient safety - Development, implementation and evaluation of an interprofessional teaching concept. GMS J Med Educ.

[21] Interprofessional Education Collaborative (2023). IPEC Core Competencies for Interprofessional Collaborative Practice: Version 3.

[22] Baker DP, Day R, Salas E (2006). Teamwork as an essential component of high-reliability organizations. Health Serv Res.

[23] King HB, Battles J, Baker DP, Alonso A, Salas E, Webster J, Toomey L, Salisbury M, Henriksen K, Battles JB, Keyes MA, Grady ML (2008). TeamSTEPPS™: Team Strategies and Tools to Enhance Performance and Patient Safety.

[24] Universitätsklinikum Bonn, Medizinische Fakultät (2022). Deutschlandweit erste Interprofessionelle Ausbildungsstation im Bereich Frauenheilkunde und Geburtshilfe am UKB.

[25] Mayring P (2015). Qualitative Inhaltsanalyse: Grundlagen und Techniken.

[26] Brock D, Abu-Rish E, Chiu C-R, Hammer D, Wilson S, Vorvick L, Blondon K, Schaad D, Liner D, Zierler B (2013). Interprofessional education in team communication: working together to improve patient safety. BMJ Qual Saf.

[27] The Joint Commission (2004). Sentinel Event Alert 30: Preventing infant death and injury during delivery.

[28] Reeves S, Perrier L, Goldman J, Della Freeth, Zwarenstein M (2013). Interprofessional education: effects on professional practice and healthcare outcomes (update). Cochrane Database of Syst Rev.

[29] Avery MD, Mathiason M, Andrighetti T, Autry AM, Cammarano D, Dau KQ, Hoffman S, Krause SA, Montgomery O, Perry A, Sankey HZ, Woodland MB, Jennings JC (2022). Improved Self-Assessed Collaboration Through Interprofessional Education: Midwifery Students and Obstetrics and Gynecology Residents Learning Together. J Midwif Women Health.

